# Antioxidant Properties of *Marrubium peregrinum* L. (Lamiaceae) Essential Oil

**DOI:** 10.3390/molecules15095943

**Published:** 2010-08-27

**Authors:** Biljana Kaurinovic, Sanja Vlaisavljevic, Mira Popovic, Djendji Vastag, Maja Djurendic-Brenesel

**Affiliations:** 1 Department of Chemistry, Faculty of Science, University of Novi Sad, Trg Dositeja Obradovica 3, 21000 Novi Sad, Serbia; 2 Clinical Center of Vojvodina, Department for Forensic Medicine, Hajduk Veljkova 7-9, 21000 Novi Sad, Serbia

**Keywords:** *Marrubium peregrinum* L., essential oils, GC-MC, antioxidant activity, lipid peroxidation

## Abstract

The antioxidant activity of *Marrubium peregrinum* essential oil, collected from three different locations [Backo Gradiste - Rimski Sanac (No.1), Novi Knezevac (No.2) and Senta (No.3)] was evaluated as free radical scavenging capacity (RSC), together with inhibition on xanthine-oxidase and effects on lipid peroxidation (LP). RSC was assessed measuring the scavenging activity of the essential oils on 2,2-diphenyl-1-picrylhydrazyl (DPPH^•^), super oxide anion (O_2_^•−^), nitric-oxide (NO^•^) and hydroxyl (OH^•^) radicals. The activities of xanthine-oxidase (XOD) was determined by the nitrite method. Effects on LP were evaluated by following the activities of essential oils in the Fe^2+^/ascorbate induction system. Experimental results indicate that the essential oil of *M. peregrinum* from Senta (No.3) exhibited the strongest inhibitory effect, as the IC_50_ value was achieved with the lowest concentration. The same result was obtained in investigation of influence of essential oil on XOD and LP. The chemical profile of essential oil was evaluated by the means of gas chromatography-mass spectrometry (GC-MS). According to the analysis, the most powerful scavenging compounds were sesquiterpene hydrocarbons (β-caryophyllene, bicyclogermacrene and germacrene-D) and oxygenated sesquiterpenes (spathulenol and caryophyllene oxide).

## 1. Introduction

For centuries, aromatic herbs and spices have been added to different foods to improve the flavor and organoleptic properties. The use of aromatic plants and spices in phytotherapy is mostly related to different activities of their essential oils, such as antimicrobial, spasmolytic, carminative, hepatoprotective, antiviral, anticarcinogenic activities, *etc.* [1,2]. Furthermore, many studies point to strong antioxidant activities of aromatic plants and their essential oils [3,4,5]. Antioxidant activities are also confirmed for most of the phenolic compounds present in different spices and herbs [3,6]. 

The enlarged Lamiaceae family contains 233 to 263 genera and 6,900 to 7,200 species. The genus Marrubium L. comprises approximately 30 species indigenous in Europe, the Mediterranean area and Asia [7]. The chemical composition of *M. peregrinum* has been extensively studied. *M. peregrinum* leaves contain no more than 0.1% of essential oil, which is of complex and variable composition [8]. Dominant monoterpenes are: α-pinene, sabinene, limonene, camphene and α−terpinolene. In a Greek sample, β-phellandrene, epi-bicyclosesquiphellandrene and bicyclogermacrene proved to be the major compounds [9], whereas the essential oil of a sample from Central Europe was rich in β-caryophyllene and its oxide, bicyclogermacrene and germacrene D [10]. In previous phytochemical investigations on *M. peregrinum*, different groups of chemicals were isolated: flavones (apigenin and luteolin) [11], flavonols (kaempferol) [12], glycosylated flavonoids, caffeic acid derivatives [13], and four diterpenoids (peregrinin, peregrinol, marrubiin and premarrubiin) [14,15].

As a medicinal plant, *M. peregrinum* has been employed against vascular diseases (antihypertensive, antispasmolitic). El Bardai *et al*. [16] have established that marrubenol inhibits contraction of rat arteries by blocking L-type calcium (Ca^2+^) channels in smooth muscle cells. Also, one of the flavonoids present in *M. peregrinum*, apigenin, was shown to express strong antioxidant effects, increasing the activities of antioxidant enzymes and, related to that, decreasing the oxidative damage to tissues [17]. 

The aim of this research was to study the chemical profile of essential oil of *Marrubium peregrinum* and the antioxidant activity *in vitro*, to assess their potential capacity as scavengers of free radicals (DPPH, O_2_^•−^, NO and OH radicals) and as inhibitors of xanthine-oxidase and lipid peroxidation. The chemical characterization of the investigated essential oils was performed by gas chromatography–mass spectrometry (GC-MS).

## 2. Results and Discussion

The content of essential oils expressed in percentages was as follows: *Marrubium peregrinum* (Backo Gradiste – Rimski Sanac; No.1), 0.11%; *Marrubium peregrinum* (Novi Knezevac; No.2), 0.09%; and *Marrubium peregrinum* (Senta; No.3), 0.14%. The results are consistent with the literature data, and a small deviation in the amount of essential oil from plants collected from the three different localities can be explained by the fact that a number of factors affects the yield of essential oils: time of collection of the plant material, maturity of plants, method of oil isolation, the herb drying method, material fermentation, agroecological conditions, *etc.*. In addition, the yield of essential oil varies with change of light intensity and medium in which the plant grows [18,19].

**Table 1:** Chemical composition of *Marrubium peregrinum* essential oil.

			Percentage		
Compounds	RI^a^	*M. peregrinum* (No. 1)	*M. peregrinum* (No. 2)	*M. peregrinum* (No. 3)	Identification method^b^
α-pinene	939	0.36	0.27	0.32	RT GC MS
camphene	950	0.10	0.10	0.13	RT^*^ MS
sabinene	972	-	0.12	0.10	RT GC MS
1-octen-3-ol	976	4.88	5.08	2.94	RT^*^ MS
β-pinene	978	0.46	0.52	0.46	RT GC MS
limonene	1030	0.98	1.12	1.20	RT GC MS
β-phellandrene	1034	0.92	0.80	0.92	RT^*^ MS
α-terpinolene	1090	0.38	0.44	0.50	RT^*^ MS
linalool	1100	0.20	0.27	0.25	RT GC MS
α-thujone	1104	2.12	2.31	3.26	RT GC MS
nonanal	1105	0.28	0.52	0.28	RT^*^ MS
β-thujone	1104	1.46	1.28	1.71	RT GC MS
geijerene	1150	3.21	4.02	3.70	RT^*^ MS
bornyl-acetate	1288	1.42	1.41	1.03	RT GC MS
carvacrol	1298	1.32	1.41	1.56	RT GC MS
pregeijerene	1303	0.56	0.77	0.54	RT^*^ MS
eugenol	1366	0.16	0.20	0.12	RT^*^ MS
α-copaene	1391	0.25	0.26	0.19	RT^*^ MS
β-bourbonene	1400	0.43	0.37	0.51	RT^*^ MS
β-cubenene	1404	0.52	0.46	0.72	RT^*^ MS
α-gurjunene	1408	3.09	3.43	3.37	RT^*^ MS
β-caryophyllene	1440	13.20	14.34	17.99	RT GC MS
(Z)-β-farnesene	1446	4.28	4.71	5.12	RT^*^ MS
α-humulene	1452	2.04	1.90	2.62	RT GC MS
(E)-β-farnesene	1456	3.71	4.37	5.08	RT^*^ MS
γ-muurolene	1494	5.59	5.56	6.26	RT^*^ MS
germacrene-D	1503	6.79	8.56	9.05	RT^*^ MS
β-ionone	1514	1.14	0.15	0.90	RT^*^ MS
bicyclogermacrene	1517	7.63	6.42	9.80	RT^*^ MS
α-muurolene	1526	0.14	0.19	0.12	RT^*^ MS
γ-cadinene	1533	-	0.10	0.10	RT^*^ MS
δ-cadinene	1540	1.33	1.61	1.66	RT^*^ MS
nerolidol	1570	0.51	0.62	0.56	RT^*^ MS
α-cadinene	1582	0.10	-	0.12	RT^*^ MS
spathulenol	1601	5.18	5.68	3.76	RT^*^ MS
caryophyllene oxide	1610	4.23	3.73	4.98	RT^*^ MS
viridiflorol	1618	0.78	0.76	0.80	RT^*^ MS
octadecane	1799	0.14	0.28	0.32	RT^*^ MS
nonadecane	1900	0.10	0.11	0.10	RT^*^ MS
undecane	1948	-	-	0.65	RT^*^ MS
dodecane	1956	1.28	1.36	0.26	RT^*^ MS
palmitic acid	1962	1.35	1.04	1.13	RT^*^ MS
heneicosane	2100	0.32	0.29	0.26	RT^*^ MS
tricosane	2299	0.72	0.66	0.70	RT^*^ MS
**Total identified**		**83.66**	**87.60**	**96.15**	
Monoterpene hydrocarbons		3.20	3.37	3.63	
Oxygenated monoterpenes		5.20	5.27	6.25	
Aromatic oxygenated monoterpenes		1.48	1.61	1.68	
Sesquiterpene hydrocarbons		49.10	52.28	62.71	
Oxygenated sesquiterpenes		11.84	10.94	11.00	
Aliphatic compounds		12.84	14.13	10.88	

*^a ^*Retention indices relative to C_9_–C_24_ *n*-alkanes on the HP 5MS column. *^b ^*(RT) comparison with pure standard retention time; (GC) gas chromatographic coelution with pure standard; (MS) mass spectrometry; (RT*) comparison of the relative retention time with those obtained from the NIST/NBS, Wiley libraries spectra, and those reported by Adams [20].

Table 1 lists the chemical components of the investigated essential oils. The total number of chemical constituents identified in essential oils was 44 for *M. peregrinum* from Senta (No.3), 42 for *M. peregrinum* from Novi Knezevac (No.2) and 41 for *M. peregrinum* from Rimski Sanac (No.1), representing 96.15%, 87.60% and 83.66% of the total oil contents, respectively. The main constituents of the essential oil of *M.* *peregrinum* from all locations were sesquiterpene hydrocarbons (β-caryophyllene, bicyclogermacrene and germacrene-D). Besides sesquiterpene hydrocarbons, oxygenated sesquiterpenes (spathulenol and caryophyllene oxide) are also present in relevant quantities. However, we must point out that the amounts of these components in essential oil from different localities are very different. Essential oil obtain from plant collected in Senta (No.3) is the richest of sesquiterpene hydrocarbons (62.71%), while oxygenated sesquiterpenes are most represented (11.84%) in essential oil from plants collected in the Rimski Sanac (No.1) area

Earlier data pertaining to *M. peregrinum* essential oil point out the persistence of two chemotypes, Greek (containing β-phellandrene, epi-bicyclosesquiphellandrene and bicyclogermacrene as major compounds, together with absence of monoterpene hydrocarbons) [9], and Central Europe, characterized by a high amount of β-caryophyllene and its oxide, bicyclogermacrene and germacrene-D [10]. The investigated essential oil, obtained from the plant material from the Pannonian plane, has a specific chemical composition and could be categorized by a chemotype which belongs to Central Europe. Also, the three populations of investigated *M. peregrinum* seem to have the same chemotype, as there are many similarities in chemical composition between the three oils, although in some cases the amounts of the corresponding components is different.

The antioxidant potential of *M. peregrinum* essential oil and pure compounds can be evaluated using numerous assays. The first step in these examinations is the screening of the potential activity by different *in vitro* tests. Each of those is based on one feature of the antioxidant activity, such as the ability of scavenging free radicals, the inhibition of lipid peroxidation, the chelating of transition metal ions (TMI), *etc.* However, in order to get relevant data, a single method for testing antioxidant activities of plant products is not recommended due to their complex composition [21]. Therefore, the antioxidant activity of the tested essential oil has been evaluated in a series of *in vitro* tests. Results of antiradical activity of *M. peregrinum* essential oil on the content of DPPH, O_2_^•−^ and NO radicals are given in Table 2. 

**Table 2:** IC_50_ values (μg/mL) of the neutralization of DPPH^•^, NO^•^ and O_2_^•−^ radicals with *Marrubium peregrinum* essential oil and BHT (as a positive control).

		IC_50_ (μg/mL)	
Source	DPPH radical	O_2_^•−^ radical	NO radical
***M. peregrinum* (No. 1)**	13.48	11.68	11.87
***M. peregrinum* (No. 2)**	13.41	16.41	13.12
***M. peregrinum* (No. 3)**	11.69	10.82	8.81
**BHT**	14.31	10.46	8.63

In the DPPH assay, the ability of the investigated essential oils to act as donors of hydrogen atoms or electrons in transformation of DPPH^•^ into its reduced form DPPH-H was investigated (Table 2). All of the assessed essential oils were able to reduce the stable, purple-colored radical DPPH to yellow-colored DPPH-H reaching 50% of reduction with IC_50_ values as follows: 13.48 μg/mL for *M. peregrinum* (Rimski sanac; No.1), 13.41 μg/mL for *M. peregrinum* (Novi Knezevac; No.2), and 11.69 μg/mL for *M. peregrinum* (Senta; No.3). Comparison of the DPPH scavenging activity of the investigated essential oils with those expressed by *tert*-butylated hydroxytoluene (BHT) (14.31 μg/mL) showed that all of the examined essential oils expressed stronger antioxidant effects. The best effect of the neutralization of DPPH radicals exhibited the essential oil collected from the Senta locality. 

In the O_2_^−•^ assay, the ability of essential oil to scavenge superoxide anion radical, was examined (Table 2). On the basis of the results obtained for the values of investigated system it can be seen that the greatest ability to neutralize superoxide anion radical has essential oil from the Senta locality (IC_50 _= 10.82 μg/mL) and this ability is a bit weaker compared to the one (IC_50 _= 10.46 μg/mL) exhibited by the synthetic antioxidant *tert*-butylated hydroxytoluene (BHT). Similar results were obtained examining the neutralization of NO radical. Again, essential oil from the Senta locality (No. 3) exhibited the strongest inhibitory effect, as the IC_50_ value was achieved at the lowest concentration (IC_50_ = 8.81 μg/mL). Inhibition of NO radicals with ether oil of *M. Peregrinum* is very significant, having in mind the ability to neutralize the superoxide anion radicals as well. Essential oil of *M. peregrinum* from Senta is especially suited in this process since it neutralizes both superoxide anion radical and NO radical. Obtained results could be explained partially by the presence of a higher amount of compounds responsible for this activity, such as sesquiterpene hydrocarbons (first β-caryophyllene) recorded in the *M. peregrinum* essential oil from Senta (Table 1). 

**Table 3:** DPPH Scavenging Active Compounds Identified by the TLC Dot-Blot Technique.

Source	Compound	Rf values
***M. peregrinum***	carvacrol	0.62
	α- and β-thujone	0.64-0.67
	bornyl acetate	0.69
	mixture of mono and sesquiterpene hydrocarbons	0.97

The comparison of control TLC analysis with the results of GCMS (Table 1) and TLC-DPPH methods accomplished the identification of the constituents most responsible for RSC. For the neutralization of DPPH^•^ radicals, the most responsible compounds were the oxygenated monoterpenes (α- and β-thujone, and bornyl acetate) and the mixture of mono- and sesquiterpene hydrocarbons (Table 3). Although found in a small amount in the essential oil of *M. peregrinum*, carvacrol (Table 1) exhibited notable scavenging activity, too. The mono- and sesquiterpene hydrocarbons showed considerable overlap when hexane is used, Therefore, in future experiments, some other more suitable should be used. These findings are correlate with the earlier published data on the antioxidant activities of the essential oil and selected essential oil components [22,23,24,25]. The obtained results also confirm the previously published data on stronger antioxidant activity of carvacrol as compared to the thymol [4]. 

**Figure 1:**
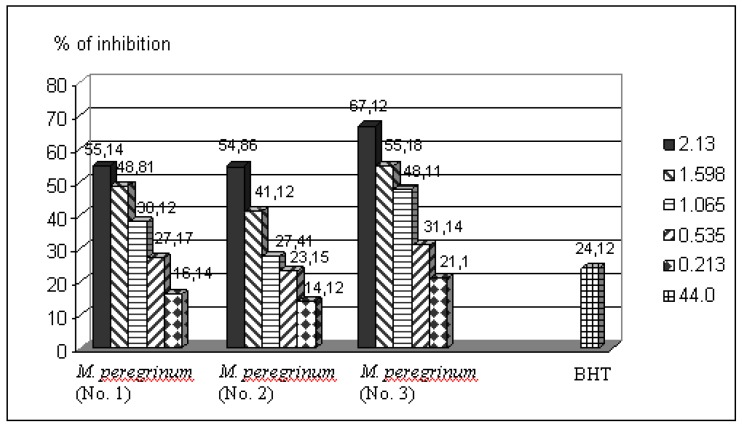
Inhibition of degradation of 2-deoxyribose by essential oils of *M. peregrinum* from three different locations, and BHT (as a positive control). The essential oils and BHT were diluted in *n*-hexane (the solvent expressed no antioxidant activity) in the deoxyribose assay.

The hydroxyl RSC of the examined essential oil (Figure 1) was measured by the deoxyribose assay. Hydroxyl radicals formed in Fenton reaction were detected by their ability to degrade 2-deoxy-D-ribose into fragments that on heating with TBA at low pH, form a pink adduct. However, this feature of the antioxidant activity of investigated essential oils has not been investigated yet. 

Generally, the examined essential oils from all three locations, except those at the lowest concentration (0.213 μg/mL), inhibited the degradation of deoxyribose more than BHT (24.12%), used as a positive control. The highest activity was shown by the essential oil of *M. peregrinum* from Senta locality (No.3), especially in concentration of 2.130 μg/mL (67.12%) and 1.598 μg/mL (55.18%). The antioxidant activities of all three essential oils were dose dependent. The results confirm the data found in the literature on which it is known that the essential oils of Lamiaceae family, which includes *M. peregrinum*, proved as a great ’’scavengers’’ of OH radicals. Probably, monoterpene ketones are responsible for this effect [5]. 

Results of activities of XOD with *M. peregrinum* essential oil are given in Table 4. Based on the shown results, it can be seen that only the essential oil from Senta (No.3) expressed stronger protective effect than BHT (19.23 μg/mL). In addition, it was found that the increase of concentration of essential oil reduce the uric acid content and the content of O_2_^−•^. This is expected because inhibition of the enzyme XOD, inhibits the creation of O_2_^−•^.

**Table 4:** IC_50_ values (μg/mL) of the activities of XOD with *Marrubium peregrinum* essential oil and and BHT (as a positive control). The essential oils and BHT were diluted in *n*-hexane (the solvent expressed no antioxidant activity) in the XOD assay.

		IC_50_ (μg/mL)		
Source	*M. peregrinum* (No. 1)	*M. peregrinum* (No. 2)	*M. peregrinum* (No. 3)	BHT
**XOD**	21.56	23.43	17.63	19.23

**Figure 2:**
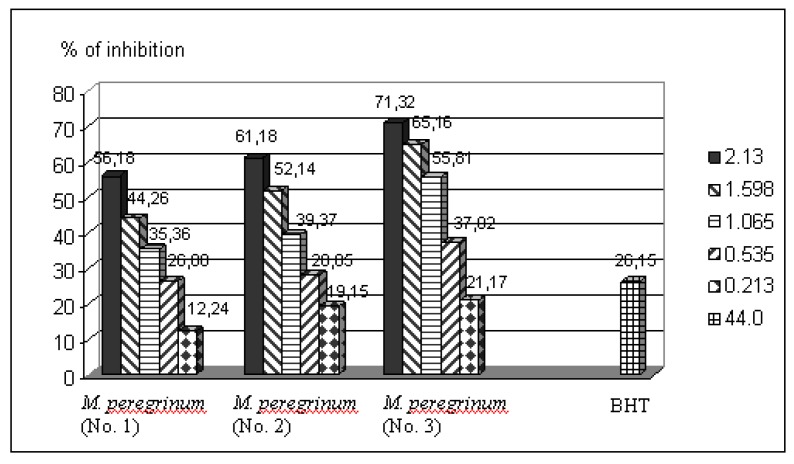
Inhibition of LP in Fe^2+^/ascorbate system of induction by essential oils of *M. peregrinum* from three different locations, and BHT (as a positive control). The essential oils and BHT were diluted in *n*-hexane (the solvent expressed no antioxidant activity) in the XOD assay.

However, if we correlate the content of uric acid and O_2_^−•^, we can observe that the values of production of superoxide are below than values of uric acid in the case of all essential oils. This means that the compounds present in the essential oils, in addition to inhibition of XOD skills, possess the ability to neutralize O_2_^−•^. As such, the essential oils from tested plant species may contain compounds that would be more effective in the treatment of gout from the widely used allopurinol, which has only the effect of XOD without additional ability to neutralize O_2_^−• ^[26]. The protective effects on lipid peroxidation (LP) of essential oils have been evaluated using the Fe^2+^/ascorbate system of induction, by the TBA-assay (Figure 2). 

Inhibition of LP was determined by measuring the formation of secondary components (mainly MDA) of the oxidative stress, using liposomes as an oxidizable substrate. In general, the examined essential oils expressed strong antioxidant capacity. The largest inhibitory activity, again, was exhibited by essential oil from plant collected at Senta locality (No.3). Solutions of all concentrations, except the most diluted (0.213 μg/mL), have exhibited a stronger protective effect (from 37.02 to 71.32% of inhibition of LP) than BHT (26.15%). The other two essential oils [from Rimski Sanac (No.1) and Novi Knezevac (No.2)], at higher concentration (from 1.065 to 2.130 μg/mL), have also exhibited more intense protective effects than BHT. 

## 3. Experimental

### 3.1. General

Plant material was collected in the Spring of 2006 from three different locations [Bačko Gradište - Rimski Sanac (No. 1), Novi Knezevac (No. 2) and Senta (No. 3)] in the Vojvodina Province, Republic of Serbia. Voucher specimens: *Marrubium peregrinum* L. 1753 No 2-2065, Bačko Gradište, Rimski Šanac, UTM34TDR24, det.: Goran Anačkov; No 2-2066, Novi Kneževac-Filić, UTM34TDS30, det.: Goran Anačkov; No 2-2067, Senta, UTM34TDR28, det.: Goran Anačkov, were confirmed and deposited at the Herbarium of the Department of Biology and Ecology (BUNS Herbarium), Faculty of Natural Sciences, University of Novi Sad.

Air-dried plant material was submitted to hydrodistillation according to Eur. Pharm. 4 [27], using *n*-hexane as a collecting solvent. The solvent was removed under vacuum. The oils were dried over anhydrous sodium sulphate and kept at +4 °C. The quantities of the essential oils were determined gravimetrically. 

### 3.2. Essential oil analysis

Qualitative and quantitative analyses of the essential oils were carried out using a Hewlett-Packard (HP, G-1800 A, GCD) gas chromatography-mass spectrometry (GC-MS) system, operating in EI mode at 70 eV, equipped with a split-splitless injector (200 °C) and a flame ionization detector (250 °C). Helium was used as a carrier gas (1 mL/min), and the capillary columns used were HP-5 MS (30 m x 0.25 mm; film thickness, 0.25 μm). The temperature programs were 60-280 °C at a rate of 3 °C/min and 60-260 °C at a rate of 3 °C/min, respectively; split ratio, 1:10. The identification of individual compounds was based on comparison of their relative retention times with those of authentic samples (Carl Roth GmbH; Karlsruhe, Germany), by coelution and MS analysis. For the components, mostly sesquiterpenes and aliphatic compounds, for which reference substances were not available, the identification was performed by matching their retention indices and mass spectra with those obtained from authentic samples and/or the NIST/NBS, Wiley Libraries spectra, and literature data [20]. 

### 3.3. In vitro experiments

Antioxidant properties of *M. peregrinum* essential oil was evaluated measuring the scavenging activity of the examined essential oil on the 2,2-diphenyl-1-picrylhydrazyl (DPPH), super oxide anion (O_2_^•^^−^), nitric-oxide (NO) and OH radicals using a Beckman DU 65 spectrophotometer. Activity of XOD and intensity of LPx in liposomes were evaluated using the same unit. 

The DPPH assay was performed as described before [28], following the transformation of the DPPH radical to its reduced, neutral form (DPPH-H). The samples (from 2.50 to 25.0 μg/mL) were mixed with 90 μM DPPH^•^ solution (1 mL) and filled up with 95% MeOH to a final volume of 4 mL. The absorbance of the resulting solutions was recorded spectrophotometrically at 515 nm after 1 h at room temperature, against the blank (with the same chemicals, except for the sample). The same procedure was repeated with *tert*-butylated hydroxytoluene (BHT) as a positive control. For each sample five replicates were recorded. The RSC in percent was calculated by following equation:

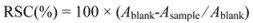


From the obtained RSC values, the IC50 values, which represented the concentrations of the essential oils that caused 50% neutralization, were determined by linear regression analysis.

Super oxide anion radicals were generated in the system xanthine/xanthine-oxidase, and the quantity of O_2_^•−^ was determined by nitrite method [26] with modifications. This system was used also in determination of the level of inhibition of xanthine-oxidase. All solutions and reagents were freshly prepared by dissolution in 0.05 M KH_2_PO_4_–K_2_HPO_4_ phosphate buffer (pH 7.4). The samples of the essential oil of *M. peregrinum* were investigated in different concentrations (from 2.50 to 25.00 μg/mL). The absorbance of the resulting solutions was recorded spectrophotometrically at 550 nm after 30 min at room temperature, against the blank (with the same chemicals, except for the xanthine-oxidase). The same procedure was repeated with BHT as a positive control. For each sample five replicates were recorded. 

Production of NO^•^ radicals was determined spectrophotometrically. NO^•^ radical generated from sodium-nitropruside (SNP) reacts with oxygen in water solution at a physiological pH to give nitrite ions. Concentration of nitrite anions was determined using Griess reagent [29]. At room temperature nitrite ions react with Griess reagent and form purple complex. The samples of the essential oil of *M. peregrinum* were investigated in different concentrations (from 2.50 to 25.00 μg/mL). The intensity of color, which is the function of the nitrite concentrations, was measured spectrophotometrically (λ = 546 nm). The absorbance of the resulting solutions and the blank (with the same chemicals, except for the sample) were recorded. For each sample, five replicates were recorded. The RSC in percent was calculated by following equation:

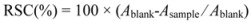


From the obtained RSC values, the IC50 values, which represented the concentrations of the essential oils that caused 50% neutralization, were determined by linear regression analysis.

Scavenging capacity of the essential oils for hydroxyl radicals was determined by monitoring the chemical degradation of 2-deoxy-D-ribose [30]. The reaction was initiated by hydroxyl radicals obtained in Fenton’s reaction [31], which yields products that react with thiobarbituric acid (TBA test). The obtained products, among which malondialdehyde (MDA) is the most important, are determined by a spectrophotometric method at 532 nm (deleted). The degradation products are the 2-thiobarbituric acid (TBA) reactive substances, which could be determined spectrophotometrically at 532 nm. All solutions and reagents were freshly prepared by dissolution in 0.05 M KH_2_PO_4_–K_2_HPO_4_ phosphate buffer (pH 7.4). For the experiment, five concentrations of essential oils were prepared: pure essential oil (2.130 μg/mL), 75% (1.598 μg/mL), 50% (1.065 μg/mL), 25% (0.535 μg/mL) and 10% (0.213 μg/mL) solution in *n*-hexane. In a test tube, pure essential oils and 75%. 50%, 25% or 10% solution in *n*-hexane (10 μL) with H_2_O_2_ (0.125 mL), FeSO_4_ (0.125 mL) and 2-deoxy-D-ribose (0.125 mL) were mixed and filled up with 0.05 M PB, pH 7.4, to a volume of 3 mL. After an incubation period of 1 h at 37 °C, the extent of deoxyribose degradation was measured by the TBA reaction. An amount of TBA reagent [1.5 mL, 10.4 mL of 10% HClO4, 3 g of TBA, and 120 g of 20% trichloroacetic acid] and 0.2 mL of 0.1 M ethylenediaminetetraacetic acid (EDTA) were added to the reaction mixture, and the tubes were heated at 100 °C for 20 min. After the mixtures were cooled, the absorbance was read against a blank (containing buffer solution instead sample) at 532 nm. A control with *n*-hexane instead of sample was also tested and expressed no activity. Five replicates were recorded for each sample. A 0.1 M concetration of BHT (44.0 μ*g*/mL) was used as a positive control. 

The absorbance reading at the end of the experiment was used for the calculation of the percentage inhibition of deoxyribose degradation by the essential oil:

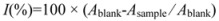



The extent of LP was determined by measuring the color of the adduct produced in the reaction between 2-thiobarbituric acid (TBA) and malondialdehyde (MDA), as an oxidation product in the peroxidation of membrane lipids, by the TBA assay [3,32,33]. The commercial preparation of liposomes ‘PRO-LIPO S’ (Lucas-Meyer) pH = 5–7 was used as a model system of biological membranes. The liposomes, 225–250 nm in diameter, were obtained by dissolving the commercial preparation in demineralized water (1:10), in an ultrasonic bath. Five concentrations of essential oils were prepared for the experiment: pure essential oil (2.13 μg/mL), 75% (1.598 μg/mL), 50% (1.065 μg/mL), 25% (0.535 μg/mL) or 10% (0213 μg/mL) solution in n-hexane. The content of the MDA (TBARS) was determined by measuring the absorbance of adduct at 532 nm. In a test tube, a suspension of liposomes (50 μL) was incubated with 0.01 M FeSO4 (20 μL), 0.01 M ascorbic acid (20 μL), and essential oil samples (10 μL) in 0.05 M KH_2_PO_4_-K_2_HPO_4_ buffer (2.90 mL, pH 7.4, 3 mL final solution). Samples were incubated at 37 °C for 1 h. LP was terminated using the reaction with 1.5 mL of TBA reagent and 0.2 or 0.1 mL of EDTA, heated at 100 °C for 20 min. After precipitated proteins were cooled and centrifuged (4000 rpm for 10 min), the content of the MDA (TBARS) was determined by measuring the absorbance of adduct at 532 nm. Analyses were compared with the commercial synthetic antioxidant BHT (0.1 M stock solution, concentration 44.0 μg/mL) as a positive control. Five replicates were performed for each sample. The control with n-hexane was also analyzed.

The percentage of LP inhibition was calculated by the following equation: 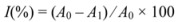



where *A*_0_ is the absorbance of the control reaction (full reaction, without the test compound) and *A*_1_ is the absorbance in the presence of the inhibitor.

### 3.4. Rapid screening for scavenging compounds of essential oils

For fast screening of essential oil compounds on RSC, the dot-blot test on thin-layer chromatography (TLC) silica gel F_254_ aluminium plates stained with the free radical DPPH^•^ was used [5]. An appropriate amount of pure essential oil (5 μL) was placed on a silica gel plate and eluted with benzene:ethyl acetate (95:5). After drying, the plates were sprayed with a 0.4 mM solution of DPPH^•^ in methanol, using a Desaga Spray Gun. Sprayed plates gave a purple background with yellow spots at the location of those compounds that possessed high RSC. Essential oil compounds responsible for scavenging activity were identified comparing the DPPH-TLC chromatogram with the control treated with vanillin–sulphuric acid spray reagent.

### 3.5. Chemicals

Thiobarbituric acid (TBA), xanthine, xanthine-oxidase, ethylenediaminetetraacetic acid (EDTA), 2,2-diphenyl-1-picrylhydrazyl (DPPH) and trichloroacetic acid were obtained from Sigma Chemicals, (St. Louis, MO, USA). 2-Deoxy-D-ribose was purchased from Aldrich. *N***-**(1-naphthyl)-ethylenediamine dihydrochloride (NEDA), silica gel F_254_ aluminium plates and *n*-hexane were obtained from Merck (Darmstadt, Germany). *tert*-butylated hydroxytoluene was obtained from Fluka, AG (Buchs, Switzerland). The commercial preparation of liposomes “PRO-LIPO S” was purchased from Lucas-Meyer (Hamburg, Germany). All chemicals used were of analytical grade.

## 4. Conclusions

In conclusion, the results of *in vitro* assays of the investigated essential oils, expressed significant RSC and protective effects on LP, which was found to be correlated to different compounds, depending on the system of examination. In particular, the investigated essential oils exhibited high RSC against DPPH, O_2_^−•^ and NO radical, which was found to correlate with the content of oxygenated monoterpenes (α- and β-thujone, and bornyl acetate) and the mixture of mono- and sesquiterpene hydrocarbons sesquiterpene hydrocarbons (primarily β-caryophyllene). Also, a very strong protective activity of the essential oils (especially the essential oil from Senta locality, No.3) in lipid peroxidation processes, especially against hydroxyl radicals formed in the Fenton reaction, was recorded. These results indicate that essential oils could serve not only as flavorings but also as safe antioxidants and antiseptic supplements in preventing deterioration of foodstuffs and beverages and pharmaceuticals. Also, consumption of food produced with natural essential oils or aromatic plant extracts (functional foods) is expected to prevent the risk of free radical dependent diseases. The use of investigated essential oils and spices could be useful not only in food and cosmetics production but also as important functional food in the prevention and treatment of various human diseases. 
